# Metabolic and volume status evaluation of hemodialysis patients with and without residual renal function in the long interdialytic interval

**DOI:** 10.1590/2175-8239-JBN-2018-0171

**Published:** 2019-01-07

**Authors:** Lenina Ludimila Sampaio de Almeida, Luís Henrique Bezerra Cavalanti Sette, Fernando Luiz Affonso Fonseca, Leila Silveira Vieira da Silva Bezerra, Francisco Hélio Oliveira, Ronaldo Roberto Bérgamo

**Affiliations:** 1 Faculdade de Medicina do ABC, Departamento de Nefrologia, Santo André, SP, Brasil.; 2 Universidade Federal de Pernambuco, Departamento de Nefrologia, Recife, PE, Brasil.; 3 Universidade Federal do Cariri, Departamento de Nefrologia, Barbalha, CE, Brasil.

**Keywords:** Renal Insufficiency, Chronic Renal, Dialysis, Hyperkalemia, Acidosis, Hyperphosphatemia, Insuficiência Renal Crônica, Diálise Renal, Hiperpotassemia, Acidose, Hiperfosfatemia

## Abstract

**Introduction:**

It is unclear whether residual renal function (RRF) in dialysis patients can attenuate the metabolic impact of the long 68-hour interdialytic interval, in which water, acid, and electrolyte accumulation occurs.

**Objective:**

to evaluate serum electrolyte levels, water balance, and acid-base status in dialytic patients with and without RRF over the long interdialytic interval (LII).

**Methodology:**

this was a single-center, cross-sectional, and analytical study that compared patients with and without RRF, defined by diuresis above 200 mL in 24 hours. Patients were weighed and serum samples were collected for biochemical and gasometric analysis at the beginning and at the end of the LII.

**Results:**

27 and 24 patients with and without RRF were evaluated, respectively. Patients without RRF had a higher increase in serum potassium during the LII (2.67 x 1.14 mEq/L, *p* < 0.001), reaching higher values at the end of the study (6.8 x 5.72 mEq/L, *p* < 0.001) and lower pH value at the beginning of the interval (7.40 x 7.43, *p* = 0.018). More patients with serum bicarbonate < 18 mEq/L (50 x 14.8%, *p* = 0.007) and mixed acid-base disorder (57.7 x 29.2%, *p* = 0.042), as well as greater interdialytic weight gain (14.67 x 8.87 mL/kg/h, *p* < 0.001) and lower natremia (137 x 139 mEq/L, *p* = 0.02) at the end of the interval. Calcemia and phosphatemia were not different between the groups.

**Conclusion:**

Patients with RRF had better control of serum potassium, sodium, acid-base status, and volemia throughout the LII.

## Resumo

**Introdução:**

Não se sabe ao certo se a função renal residual (FRR) de pacientes dialíticos pode atenuar o impacto metabólico do maior intervalo interdialítico (MII) de 68 horas, no qual ocorre acúmulo de volume, ácidos e eletrólitos.

**Objetivo:**

Avaliar os níveis séricos de eletrólitos, balanço hídrico e status ácido-básico de pacientes dialíticos com e sem FRR ao longo do MII.

**Metodologia:**

Tratou-se de estudo unicêntrico, transversal e analítico, que comparou pacientes com e sem FRR, definida como diurese acima de 200 mL em 24 horas. Para tal, os pacientes foram pesados e submetidos à coleta de amostras séricas para análise bioquímica e gasométrica no início e fim do MII.

**Resultados:**

Foram avaliados 27 e 24 pacientes com e sem FRR, respectivamente. Pacientes sem FRR apresentaram maior aumento de potássio sérico durante o MII (2,67 x 1,14 mEq/L, *p* < 0,001) atingindo valores mais elevados no fim (6,8 x 5,72 mEq/L, *p* < 0,001); menor valor de pH no início do intervalo (7,40 x 7,43, *p* = 0,018), maior proporção de pacientes com bicarbonato sérico < 18 mEq/L (50 x 14,8 %, *p* = 0,007) e distúrbio ácido-básico misto (70,8 x 42,3 %, *p* = 0,042), além de maior ganho de peso interdialítico (14,67 x 8,87 mL/kg/h, *p* < 0,001) e menor natremia (137 x 139 mEq/L, *p* = 0,02) no fim do intervalo. A calcemia e fosfatemia não foram diferentes entre os grupos.

**Conclusão:**

Pacientes com FRR apresentaram melhor controle dos níveis séricos de potássio, sódio, status ácido-básico e da volemia ao longo do MII.

## Introduction

Dialysis patients have a higher risk of morbidity and mortality than the general population ¹. This risk appears to be increased in the long interdialytic interval (LII), a 68-hour period without hemodialysis (HD), to which patients undergoing conventional HD treatment three times a week are submitted and in which there is a greater number of hospitalizations and cardiovascular events^2^^,^ 3. Probably, this fact stems from the greater accumulation of uremic toxins, acids, electrolytes, especially potassium, and fluids in this time interval^4^^-^ 8. Besides that, removal of these elements in the first HD session subsequent to the LII, occurs more intensely, resulting in abrupt fluctuations of electrolytes and greater hemodynamic instability^9^.

In addition, the presence of residual renal function (RRF), which can be defined as a 24-hour urine output greater than 200 mL, is associated with a lower risk of morbidity and mortality in dialysis patients^10^^,^ 11. In fact, patients with RRF have higher excretion of sodium and water, with consequent lower interdialytic weight gain (IDWG) and more adequate blood pressure (BP) levels^12^^,^ 13. Besides that, they have better control of serum potassium, phosphate, and bicarbonate levels 14 ^-^ 16.

Since dialytic patients with RRF have a greater ability to excrete electrolytes, acids, and fluid compared to patients without RRF, they are likely to have less body accumulation of these elements during the LII and exhibit a better metabolic and hemodynamic profile in this period. However, there are few studies comparing patients with and without RRF regarding serum electrolyte levels (sodium, potassium, calcium, and phosphate), acid-base status (pH, pCO_2_, and bicarbonate) and water balance, specifically throughout the LII. We understand that obtaining these data is important, since it can encourage practices aimed at the preservation of RRF, in addition to promoting therapeutic strategies to minimize the deleterious effects of the LII in the population of patients without RRF. Thus, this study aims to evaluate the variation of electrolytes, acid-base status, and volume status over the LII in patients with and without RRF.

## Patients and methods

### Patients

The study was performed with patients submitted to HD at the Raimundo Bezerra Hemodialysis Unit in the city of Crato, Ceará. This unit has 289 patients distributed in three shifts: on Mondays, Wednesdays, and Fridays (MWF); and Tuesdays, Thursdays, and Saturdays (TTS). Patients were selected from the first and second shifts of MWF and the first shift of TTS. Patients on HD for less than three months, younger than 18 years, who had less than 12 hours of prescribed dialysis per week, and those unable to measure urinary volume were excluded from the study. The patients used polyethersulfone membrane dialyzers: Elisio-19 H and 21 H (Japan, 2016) and commercial dialysate with the following concentrations: sodium: 138 mEq/L, potassium: 2 mEq/L, calcium: 3.5 mEq/L, and bicarbonate: 32 mEq/L. The informed consent form was obtained from all patients. The study was conducted in accordance with the principles of the Declaration of Helsinki and approved by the Ethics Committee of the Faculty of Medicine of ABC - Santo André / São Paulo.

### Study design

This was a single-center, cross-sectional, and analytical study with the objective of evaluating the metabolic and hemodynamic changes over the LII in dialytic patients with and without RRF. In May 2017, 128 dialysis patients who met the inclusion criteria were questioned about the presence of RRF, defined as a 24-hour urinary output >200 mL. Of these, 42 patients reported the presence of RRF and 86 its absence. Thirty patients from each group were randomly selected by lot.

### Data collection

Patients with RRF were instructed to collect 24 hour urine volume to measure urea and creatinine clearances. For those on a MWF schedule, the urine collection started from the time they first emptied their bladder on Sunday until the same time on Monday. Patients on a TTS schedule performed the same procedure from Monday to Tuesday. Blood samples were collected at the beginning and at the end of the LII. Samples obtained after the last HD session prior to LII were collected at the end of the fourth hour of dialysis, through the arterial blood line of the HD circuit; the samples from the end of the LII were collected prior to the HD session, through the arterial line of the circuit after its connection to the patient. Two 3-mL samples were collected at each time point; one for biochemical analysis, in which urea, sodium, potassium, calcium, and phosphate were measured; and another, for blood gases, in which pH, bicarbonate, and pCO_2_ were measured. In order to determine plasma creatinine clearance, a serum creatinine measurement was performed at the end of the LII. Serum albumin was measured at the end of the interval for correction of serum calcium.

Information on the etiology of renal disease, medications used and KT/V were obtained through the patients' electronic records.

Patients were weighted at the beginning and at the end of the LII and IDWG was calculated from the difference between the two values. Blood pressure was measured at the end of the LII using mercury sphygmomanometer in sitting position after a 10-min rest period by trained professionals.

### Biochemical analysis

Biochemical samples were analyzed on Vitros 5600 Integrated System - Ortho Clinical Diagnostics (Johnson & Johnson, New Jersey, USA) using the Reflectance Spectrometer (Dry Chemistry) methodology.

Blood gases were analyzed on Gen Premier blood gas analyzer (Instrumentation Laboratory, Massachusetts, USA) using the potentiometric method. The study followed good laboratory practices.

### Calculations and definitions

Variation in electrolytes (potassium, sodium, calcium, and phosphate) and gasometric parameters (pH, pCO_2_ and bicarbonate) were calculated by the difference between the values found after and before the LII.

The KT/V value recorded in the electronic medical record was obtained using the Daugirdas formula^17^ in the month preceding the sample collection and values greater than or equal to 1.2 per session were considered adequate^18^.

Urea and creatinine clearances were calculated using the formula:

#### Urinary urea or creatinine concentration (mg/dL) x Urinary volume (mL)

Urea or creatinine serum concentration (mg/dL) x 1440

Urinary urea and creatinine were obtained from the 24-hour urine collection and serum urea and creatinine from serum samples collected at the end of the interval. The values obtained were corrected to 1.73 m^2^ of body surface area and the mean urea and creatinine clearance were calculated.

Serum calcium level was corrected according to serum albumin by the formula:

Corrected (Ca) = Measured total (Ca) + (0.8 x (4 - serum albumin))

The expected pCO_2_ was estimated for each patient from the bicarbonate measured in blood gas analysis through the formula HCO_3_ + 15^19^. For patients with fistula, blood gases in which the oxygen saturation was > 95% were consider for analysis and PCO_2_ values that were up to 5 mmHg higher or lower than the expected PCO_2_ were considered adequate. For patients with catheter, 4 mmHg was subtracted from the PCO_2_ measured in blood gas analysis, since central venous PCO_2_ is about 4 mmHg higher than arterial PCO_2_, and resulting values that were up to 5 mmHg higher or lower than the expected PCO_2_ were considered adequate^5^.

NPCR (normalized protein catabolic rate) was calculated by urea variation in the LII, taking into account urinary urea in the case of patients who had RRF^20^. As diuresis was collected in only one day of the interval, the same value of urine urea for the day not collected was considered for calculation. Values higher than 1.2 g/kg per day were considered adequate^21^.

IDWG <13 mL/kg/h, pre-HD systolic BP between 130 and 159 mmHg and diastolic BP between 60 and 89 mmHg were considered adequate^22^^,^ 23.

### Statistical analysis

Descriptive statistics of central tendency, mean and standard deviation were used for all continuous variables, and frequency distribution for categorical variables. The t-test for independent samples was conducted to evaluate possible differences between continuous variables of the two groups, and the chi-square association test (linear by linear) to verify possible differences between categorical variables. Values of *p* < 0.05 were considered significant. The Jasp program (Free Version 0.8.5.0) was used in all analyses.

## Results

Of the 30 patients initially allocated to each group, 7 (2 with RRF and 5 without RRF) were excluded because they did not perform 12 hours of HD during the week of the study. One patient in the RRF group was excluded because he did not collect diuresis for analysis and 1 patient in the RRF group was excluded because he was in another city during the collection period. We then evaluated 27 and 24 patients with and without RRF, respectively.

Baseline characteristics of the patients are shown in Table 1. The measured mean urea and creatinine clearance was 3.6 mL/min in the RRF group. The groups were similar, even in relation to Kt/V and type of vascular access for HD, presenting statistically significant difference only in relation to dialysis vintage (2.1 x 7.2 years in the groups with and without RRF, respectively; *p* < 0.001) and to the amount of calcium carbonate tablets used, higher in the group without RRF.

**Table 1:** Clinical characteristics of patients on hemodialysis in the city of Crato, CE

	With residual function	Without residual function	*p*
N	27	24	
Shift			
MWF (%)	81.5	88	0.515†
Age (years)	46. 81 ± 16.38	52.50 ± 17.16	0.232#
Male (%)	48.5	70.8	0.100†
Residual diuresis (mL)	930 ± 423.3		
Urea clearance (mL/min)	2.61 ± 2.13	-	
Creatinine clearance (mL/min)	5.42 ± 3.13		
Mean urea and creatinine clearance (mL/min)	3.68 ± 2.12		
Vascular access arteriovenous fistula (%)	87.5	95.8	0.739†
Base Disease (%)			0.327†
Hypertension	37	20.8	
Diabetes	3.7	4.2	
Glomerulonephritis	11.1	16.7	
ADPKD	14.8	8.3	
Obstructive Uropathy	18.5	12.5	
Unknown	14.8	37.5	
Hemodialysis vintage (years)	2.10 ± 1.91	7.20 ± 3.13	< 0.001#
KT/V	1.31 ± 0.36	1.22 ± 0.28	0.36#
Adequate KTV (%)	74.1	70.8	0.79†
Antihypertensive drugs that cause Hyperkalemia			0.395†
(ACEI / ARB / Spironolactone / Beta-Blocker) (%)			
None	33.3	50	
One	59.3	41.7	
Two	7.4	4.2	
Three	0.0	4.2	
Four	0.0	0.0	
Diuretic (%)	18.5	8.3	0.291†
Other Antihypertensives (%)			0.729†
None	66.7	62.5	
One	22.2	25	
Two	7.4	12.5	
Three	3.7	0.0	
Erythropoietin (%)			0.528†
Did not use	7.4	12.5	
≤ 4000 U / week	44.4	25	
Between 4000 and 8000 U / Week	11.1	16.7	
> 8000 U / week	37	45.8	
Sevelamer (%)			0.873†
Did not use	66.7	66.7	
1 tablet/day	0.0	0.0	
2 tablets /day	14.8	12.5	
3 tablets/day	11.1	16.7	
≥ 4 tablets/day	7.4	4.2	
Calcium carbonate (%)			0.018†
Do not use	74.1	62.5	
1 tablet /day	14.8	0.0	
2 tablets /day	7.4	4.2	
3 tablets /day	3.7	4.2	
≥ 4 tablets/day	0.0	29.2	
Calcitriol			0.476†
Did not use	77.8	66.7	
1 tablets/day	14.8	16.7	
2 tablets/day	3.7	12.5	
3 tablets /day	0.0	4.2	

† X2 (linear by linear)

# t-test for independent samples

**Figure 1:**
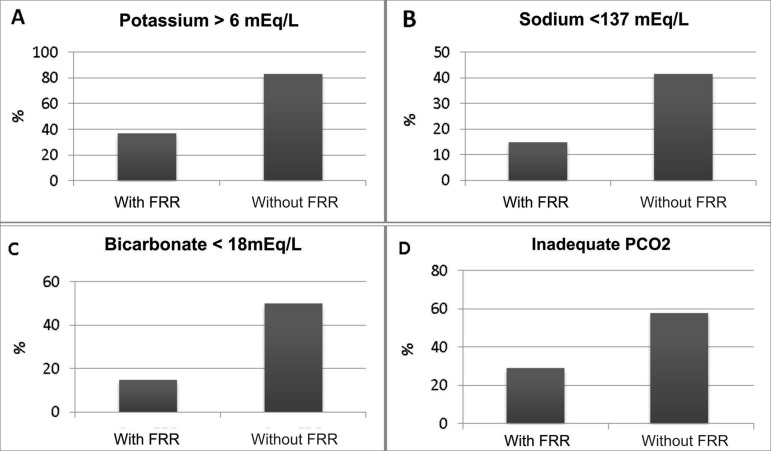
Hydroeletrolytic and acid-base disorders after the long interdialytic interval according to residual renal function

In relation to electrolyte changes, patients without RRF, despite starting from similar serum potassium values, presented a significantly higher increase (2.67 x 1.14 mEq / L, *p* < 0.001) of the electrolyte serum level throughout the LII, culminating in higher values at the end of the study period (Table 2 and Figure 1 A). In addition, they exhibited lower serum sodium levels at the end of the LII (Table 2), with a higher proportion of patients with natremia below 137 mEq/L (Figure 1 B), although they had similar serum levels at the beginning of the interval. Calcemia and phosphatemia were similar in the groups with and without RRF over the LII (Table 2). There was no difference between the groups in relation to the proportion of patients with normal phosphatemia (between 2.5 and 4.5 mg/dL) at the end of the interval (44.4 x 66.6%, *p* = 0.11).

**Table 2:** Variation of electrolytes over the LII according to RRF

Electrolyte	With RRF	Without RRF	*p*
	n= 27	n=24	
Potassium (meq/L)			
Beginning of the LII	4.58 ± 0.91	4.12 ± 0.67	0.08#
Mean variation	1.14 ± 1.26	2.67 ± 1.23	< 0.001#
End of the LII	5.72 ± 0.96	6.8 ± 0.67	< 0.001#
Sodium (mmol/L)			
Beginning of the LII	139.03 ± 5.14	137.87 ±2.99	0.337#
Mean variation	0.0 ± 5,1	- 0.8 ± 3.0	0.542#
End of the LII	139.03 ± 3.00	137.08± 2.78	0.020#
Phosphate (mg/dL)			
Beginning of the LII	3.73 ± 0.84	4.43 ± 1.69	0.064#
Mean variation	1.24 ± 1.61	1.12 ± 1.62	0.784#
End of the LII	4.98 ± 1.54	5.55 ± 1.90	0.241#
Corrected Calcium (mg/dL)			
Beginning of the LII	10.85 ± 1,09	10.70 ± 0.67	0.55#
Mean variation	- 2.11 ± 0.95	-1.90 ± 1.50	0.68#
End of the LII	8.74 ± 0.62	8.73 ± 1.22	0.96#

# t-test for independent samples.

Regarding acid-base status, the group without RRF had a lower pH value at the beginning and a trend towards a lower value at the end of the interval (Table 3). Although serum bicarbonate level was similar between the groups over the interval (Table 3), the group without RRF had a higher proportion of patients with bicarbonate values lower than 18 mEq/L at the end of the LII (Figure 1C). There was no difference between the groups with and without RRF in relation to PCO_2_ values at baseline and at the end of the interval (Table 3). Mean PCO_2_ variation was also similar between the groups (Table 3); however, in the non-RRF group there was a higher proportion of patients with pCO_2_ values inadequate for the bicarbonate values found, that is, with mixed acid-base disorder at the end of the LII (Figure 1D). The respiratory disorder was found to be respiratory acidosis (pCO_2_ exceeding 5 mmHg the expected value) in 100% of patients with mixed acid-base disorder in the group without RRF and in 91.7% in the group with RRF (only one patient presented respiratory alkalosis associated with metabolic acidosis).There was no evidence of metabolic alkalosis at the end of the LII in any of the study patients.

**Table 3:** Variation of acid-base status over the LII according to RRF

	With RRF	Without RRF	*p*
	n= 27	n=24	
pH			
Beginning of the LII	7.43 ± 0.47	7.40 ± 0.04	0.018#
Mean variation	-0.12 ± 0.05	-0.12 ± 0.08	0.940#
End of the LII	7.30 ± 0.05	7.27 ± 0.06	0.073#
Bicarbonate			
Beginning of the LII	26.62 ± 2.50	26.00 ± 2.40	0.372#
Mean variation	-6.71 ± 3.52	- 6.76 ± 3.48	0.959#
End of the LII	19.91 ± 2.85	19.24 ± 2.84	0.403#
pCO_2_			
Beginning of the LII	39.80 ± 3.99	41.93 ± 5.47	0.116#
Mean variation	- 0.60 ±4.08	-0.88 ± 4.70	0.821#
End of the LII	39.19 ± 6.24	41.04 ± 4.02	0.221#

# t-test for independent samples

Patients without RRF had higher IDWG over the LII (14.67 x 8.87 mL/kg/h, *p* < 0.001), as well as higher proportion of patients with inadequate IDWG, although blood pressure levels did not reach a statistically significant difference between the groups (Table 4).

**Table 4:** Hemodynamic changes, nPCR and albumin according to RRF

	With residual function	Without residual function	*p*
	n=27	n=24	
Interdialytic weight gain (mL/kg/h)	8.87 ± 4.77	14.67 ± 4.80	< 0.001#
Adequate interdialytic weight gain (%)	70.3 ± 4.7	37.5 ± 4.8	0.019†
SBP at the end of the LII (mmHg)	141 ± 21.1	151 ± 24 .7	0.125#
DBP at the end of the LII (mmHg)	81 ± 10.59	85 ± 13.8	0.319#
Adequate BP at the end of the LII (%)	51.5	37.5	0.197†
nPCR (g/kg/day)	0.91 ± 0.33	0.86 ± 0.26	0.53#
Adequate nPCR (%)	33.3	29.2	0.57†
Albumin	3.89 ± 0.41	4.08 ± 0.37	0.09#

Data presented as mean ± standard deviation or %

# t-test for independent samples

† X2 (linear by linear)

SBP, systolic blood pressure; DBP, diastolic blood pressure; nPCR, normalized protein catabolic rate.

There was no difference between serum albumin levels and npcr between the two groups (Table 4).

## Discussion

Patients with RRF had more adequate serum sodium and potassium levels, better acid-base status and lower IDWG over the LII when compared to patients without RRF. They also had a lower dialysis vintage, as expected, since loss of RRF occurs with the passage of years after initiation of dialysis therapy^15^. To the best of our knowledge, this is the first article comparing the entire interdialytic interval in patients with and without residual renal function in relation to electrolytes and acid-base status (pH, pCO_2_, and bicarbonate).

Regarding potassium, the findings were similar to those found in prior studies. In fact, a 2009 Egyptian study comparing serum potassium levels of 400 dialysis patients with and without RRF at the beginning and end of the interdialytic interval also found higher potassium levels in the non-RRF group at the end of the interval (5.89 x 5.12 mEq/L, *p* < 0.001), although, unlike our study, these authors evaluated patients in different dialytic intervals^14^. In contrast, potassium levels at the beginning of the interval were higher in the non-RRF group (4.29 x 3.60 mEq/L, *p* < 0.001), while in our study the values measured at this time were similar, which could have resulted from factors related to HD efficiency, not evaluated in the aforementioned study. The authors also did not compare the mean values of potassemia variation over the interval between the groups, in contrast to our study that found significantly higher values in the group without RRF.

Vilar et al.^12^ compared pre-HD serum potassium obtained in monthly collections from 650 English patients on hemodialysis with and without RRF, during six months after onset of HD. The authors found significantly higher values in the group without RRF in the majority of months in which the electrolyte was dosed (5.37 x 5.10 mEq/L, *p* = 0.005, in the month with the highest serum level in both groups). However, unlike our study, there was no evaluation of the entire interdialytic interval and the criterion used to define RRF was the presence of a urea clearance greater than 1 mL/min/1.73 m^2^.

It should be noted that in the studies mentioned above, serum potassium levels at the end of the interdialytic interval were lower than in our study. One of the possible explanations for this is that these data, unlike ours, were not obtained exclusively after the LII, when serum potassium levels are generally higher than in the middle of the week. In this regard, Yusuf et al.^24^ found a 2 to 2.4-fold higher prevalence of hyperkalemia after the LII when compared to the short interval, in a cohort of American dialysis patients between 2007 and 2010. However, in the data obtained from DOPPS (Dialysis Outcomes and Practice Patterns Study, which assessed data from 20 countries between 1996 and 2015), the difference between serum potassium levels obtained after the long and short interdialytic interval ranged from only 0.01 mEq/L in China to 0.19 mEq/L in Germany^25^.

It is important to emphasize two factors that demonstrate the great vulnerability of patients without RRF during the LII. The first is related to the fact that the morbimortality associated with hyperkalemia in patients on HD is even more significant when potassium values are above 6 mEq/L, which occurred in 83% of patients without RRF in our study^24^^,^ 25. The second was the large variation of serum potassium levels in these patients throughout the LII, since, although starting from values similar to those of patients with RRF at the beginning of the interval, they reached significantly higher values at the end, which exposed them to a higher electrolyte gradient during HD session and increased the risk of adverse events^9^. Although we have not evaluated serum potassium after the rebound effect occurred within 6 hours after the end of dialysis, this effect is known to occur more intensely the higher the pre-dialytic serum potassium levels are, which could make patients without RRF even more vulnerable to it.^26^

Regarding acid-base balance disorders, patients with RRF presented higher pH values at the beginning of the LII, maintaining this trend throughout the interval, although without statistical significance at the end of the period. Nonetheless, serum bicarbonate levels did not differ during the interval between the groups. In contrast, Suda et al.^16^, when comparing 41 patients from a dialysis center in Japan with and without RRF at the end of the LII, found significantly higher values of bicarbonate in the RRF group (19.5 x 18.2 mEq/L; *p* = 0.032), although they did not evaluate other gasometric parameters. However, although the mean bicarbonate value was not different between the groups in our study, the prevalence of patients with serum bicarbonate < 18 mEq/L at the end of the LII was significantly higher (50 x 14.8%) in the group without RRF, emphasizing that this range of values was associated with higher mortality^27^. Similarly, Raikou et al.^28^ divided 52 dialytic patients into two groups according to their serum bicarbonate levels higher or lower than 22 mEq/L and found a positive association (log rank = 3.9, *p* = 0.04) between the absence of RRF and lower values of bicarbonate.

However, due to the conflicting results of studies on bicarbonate values and mortality in dialysis patients, it is believed that their joint analysis with pH and pCO_2_ leads to a more adequate understanding of .patient*s*' acid-base status due to the high prevalence of mixed disorders in this population^26^^,^ 29. Thus, our study showed a significantly higher proportion of patients with mixed disorder in the group without RRF (57.7 x 29.2%), represented by respiratory acidosis associated with metabolic acidosis in all patients in this group. This fact could show the lower capacity of these patients to compensate for variations of bicarbonate, eliminating CO_2_ through respiration.

Our study suggests that patients without RRF are more likely to have lower bicarbonate levels (< 18 mEq/L) and inadequate respiratory response to metabolic acidosis. A possible explanation for this would be pulmonary congestion, since these patients also present a higher IDWG, as observed in our study.

We found no difference between nPCR and serum albumin in the two groups evaluated, contrary to literature findings, where RRF is associated with better nutritional parameters, possibly due to our small sample size.^16^ Patients in both groups had a mean nPCR value below the adequate value, although it was higher than the critical value (0.8 g/kg per day); the mean serum albumin was higher than the recommended minimum value (3.8 g/dL) and the mean Kt/V value was adequate in both groups. It is possible that the low nPCR values found were related to the mean bicarbonate levels of the two groups considered below the recommended level (< 22 meq/L) or related to social issues or comorbidities not evaluated in our study.^18^

Serum phosphate levels were not different over the LII between the two groups. These results differ from those found in most studies in the literature^15^^,^ 30 ^,^ 31, although not in all^31^. It is possible that the small sample size as well as urea and creatinine clearance values of our study contributed to these findings. Indeed, Penne et al.^31^, in 2010, when evaluating the phosphatemia of 552 patients with and without RRF after the LII, found a higher proportion of patients with normal serum phosphate levels only in the subgroup who had mean urea and creatinine clearance higher than 4.13 mL/min compared to the subgroup of patients without RRF (64 x 48%, odds ratio 2.4, < 0.005). When comparing the subgroup with mean clearance below 4.13 mL/min, as in our study, with the subgroup of patients without RRF, there was no significant difference between the groups. Similarly, Rhee et al.^15^ evaluated 77 patients from a Korean dialysis center, showing lower phosphatemia values in the RRF group compared to the non-RRF group (4.32 x 5.32; *p* = 0.017), but the mean urea and creatinine clearance in the RRF group was also higher than ours (6.4 x 3,6 mL/min).

Serum calcium levels were similar in the two groups during the interval. The results in the literature are controversial in this regard. Indeed, Shin et al.^32^, when comparing patients with urea clearance higher and lower than 0.9 mL/min in three Korean HD units, also found no difference between the groups for serum calcium levels (8.7 x 8.6 mg/dL, *p* = 0.92). On the other hand, Wang et al.^30^ performed a single-center study with 134 Chinese patients who found lower calcium values in the RRF group (9.1 x 9.8 mg/dL; *p* < 0.001). The great variation of calcemia in dialysis patients, influenced by factors other than renal excretion, could explain these different results^32^.

As expected and reported by other studies, we found a significantly higher IDWG in patients without RRF when compared to patients with RRF, in addition to a higher prevalence of patients with inadequate weight gain (> 13 mL/kg/h) in the first group 12 ^,^ 31. It is known that this excessive fluid accumulation, more pronounced in the LII, is associated with both long-term cardiovascular morbidity, probably because it increases the risk of left ventricular hypertrophy, and to the probability of hypotension and cardiovascular instability during HD session 9 ^,^ 10.

Although there is a relationship between IDWG and BP increase in the LII, this relationship is not linear^34^. Thus, we did not find a difference between the two groups regarding mean BP, proportion of patients with adequate BP levels at the end of the interval, and amount of antihypertensive drugs used, findings similar to those reported in the literature^12^^,^ 30 ^,^ 32.

Finally, in relation to serum sodium levels, patients without RRF presented significantly lower values at the end of the LII, which could be associated with the higher IDWG evidenced in patients of this group^35^^,^ 36. However, Abalate et al.^37^, when evaluating 98 dialysis patients from a Spanish center in relation to natremia, found no difference in the proportion of patients who had RRF in the groups divided by serum sodium: na < 138 mEq/L, between 138 and 140, and > 142 mEq/L (25, 33.3, and 41.7%, respectively; *p* = not significant), although natremia had a negative relation with IDWG. In this study, natremia was not specifically evaluated in the LII, the period in which there is a greater difference in IDWG and possibly in natremia between patients with and without RRF. Nonetheless, lower sodium values are associated with higher mortality in dialysis patients in several studies, even when adjusted for other possible confounding factors, such as higher IDWG, heart failure, and RRF; natremia below 137 mEq/L was associated with greater risk of negative outcomes in the study by Hecking et al.^37^, highlighting the greater proportion of patients in this range of values in the group without RRF (45 x 11%) in our study^35^^,^ 38.

Our study had limitations. One of them was the small sample size, which probably made it difficult to obtain statistically significant results in some analyzed variables. In addition, we did not evaluate patients' diet in the studied interval nor their comorbidities, factors that could have interfered in the results. Although residual renal function can be measured through the mean of 24 hour urea and creatinine clearance^15^, the ideal would be to collect diuresis throughout the entire interdialytic interval. Another point is that we defined FRR as a urinary volume greater than 200 mL according to some studies, although others consider 100 mL as cutoff point, which could also interfere with the results.

## Conclusion

Patients without residual renal function had greater accumulation of potassium and interdialytic weight gain throughout the long interdialytic interval, in addition to lower natremia and higher prevalence of mixed acid-base disorders at the end of the period when compared to patients with residual renal function. More studies are needed about the long interdialytic interval, with a larger number of patients, in order to confirm these data and to find other possibly associations not evidenced by the small sample size.
